# Systems-Pharmacology-Based Identification of Antitumor Necrosis Factor Effect in Mimeng Flower Decoction for the Treatment of Diabetic Retinopathy

**DOI:** 10.1155/2019/5107103

**Published:** 2019-11-19

**Authors:** Yingzi Li, Ying Huang, Changsen Tu

**Affiliations:** Eye Hospital, Wenzhou Medical University, WenZhou 325000, China

## Abstract

The traditional Chinese medicine of Mimeng flower decoction (MFD) is effective in treating diabetic retinopathy (DR), but the mechanism is still unclear. This study aims at investigating the mechanism of MFD in treating DR. First, active compounds in MFD were filtered out by the systems pharmacology method and used as bait to fish potential targets. The common genes between the targets and DR-related genes were selected to construct the compound-target-disease network and identify the network hub gene as a key gene. Molecular docking was simulated to assess the binding affinity of active compounds towards the gene protein. Streptozotocin- (STZ-) induced diabetic rat model was administered to evaluate the efficacy of MFD in treating DR and its effects on retinal gene expression. Finally, 53 active compounds were screened out from the seven herbs in MFD, with a total of 136 targets. After intersecting with 210 DR-related genes, 21 common genes were applied to construct the network, and tumor necrosis factor (TNF) was identified as the hub gene. The active compounds of acacetin, kaempferol, luteolin, and quercetin showed a good binding affinity towards TNF (C-score ≥ 4). In diabetic rats, MFD treatment reversed the retinal impairment and decreased retinal TNF expression significantly. In conclusion, this study adopted the method of systems pharmacology to screen out active compounds and construct the compound-target-disease network and found that MFD could ameliorate DR by downregulating the network hub gene of TNF.

## 1. Introduction

Diabetic retinopathy (DR) is one of the most common and serious complications in diabetes mellitus (DM), characterized by chronic and progressive retinal microvascular lesions [1]. It has become the leading cause of vision loss and blindness among working adults in developed countries [2]. Almost all patients of type 1 DM (T1DM) and 60% of T2DM patients will develop visual impairment after a disease duration of 15∼20 years [3]. DR greatly lowers the life quality of diabetic patients and causes a heavy burden to the society.

Traditional Chinese medicine (TCM) has been widely used in the treatment of DR, which was built on the medical practices of ancient Chinese scholars for more than 2500 years. According to the syndrome differentiation of TCM, DR is characterized by blood stasis and the deficiency of “qi” and “yin” [4]. TCM formulas usually comprise multiple herbs, which contain multiple active compounds and mediate various biological processes. Thus, TCM formulas exacted an outstanding role in the treatment of complicated diseases. Mimeng flower decoction (MFD) composes of seven herbs: Mimenghua (Latin name: *Buddlejae Flos*), Huanglian (*Coptidis Rhizoma*), Rougui (*Cinnanmomi Cortex*), Huangqi (*Hedysarum Multijugum Maxim.*), Nvzhenzi (*Fructus Ligustri Lucidi*), Wumei (*Mume Fructus*), and Yimucao (*Leonuri Herba*). According to the TCM theory, the decoction is characterized with invigorating blood circulation and tonifying “qi” and “yin,” and thus, it was thought to be effective in treating DR. Although the efficacy has been implicated in several clinical trials, the bioactive ingredients and potential targets are still unclear [5–10].

**Figure 1:**
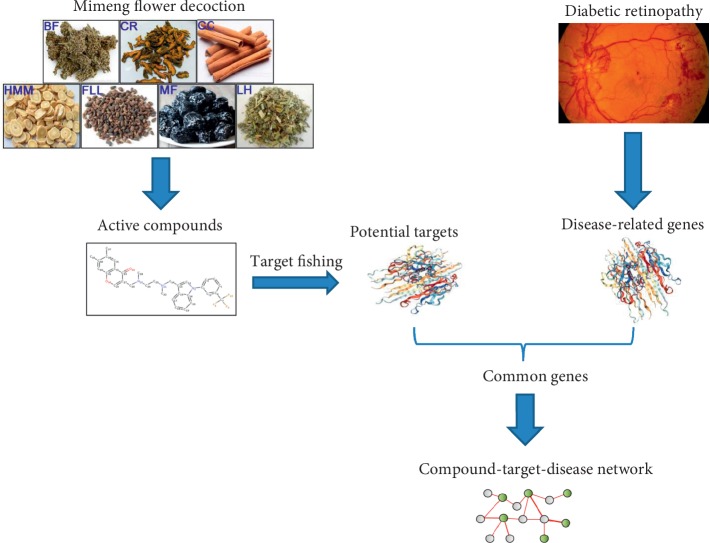
Flowchart of the systems pharmacology method in this study. BF, *Buddlejae Flos*; CR: *Coptidis Rhizoma*; CC, *Cinnanmomi Cortex*; HMM, *Hedysarum Multijugum Maxim.*; FLL, *Fructus Ligustri Lucidi*; MF, *Mume Fructus*; LH, *Leonuri Herba*.

In this study, we took a novel systems-pharmacology-based method to identify the molecular mechanism of MFD in treating DR. First, we filtered out the potential active components using the pharmacokinetic models. Then, the component-target and disease-target network was integrated, and network hub genes were experimentally validated (Figure 1).

## 2. Methods

### 2.1. Meta-Analysis of the Efficacy of MFD in Treating DR

Clinical trials focusing on the efficacy of MFD in treating DR were retrieved from the databases of PubMed, China Knowledge Resource Integrated Database (CNKI), and China Wanfang Database, using the keywords “Mimenghua” or “Miming flower” or “Buddlejae.” Relative risks (RR) with 95% confidence intervals (CI) were used to report the risk estimates following the Mantel–Haenszel method and random-effects model. The meta-analysis was performed by Review Manager 5.3.

### 2.2. Screening Active Components in MFD and the Targets

Traditional Chinese Medicines for Systems Pharmacology Database (TCMSP, https://5th.tcmspw.com/tcmsp.php) were searched for chemical ingredients of the seven herbs in MFD. Then, we screened the active constituents according to the ADME (absorption, distribution, metabolism, and excretion) principle, namely, drug-likeness (DL) ≥0.18 and oral bioavailability (OB) ≥30%. Subsequently, these active components were used as bait to fish for the targets in TCMSP, which integrated the databases of DrugBank and HIT and the SysDT model [11]. The gene names were converted into official gene symbols using an R-script linking the website of PubMed (https://www.ncbi.nlm.nih.gov/gene/), and we only included human genes.

### 2.3. Screening DR-Related Genes

DR-related genes were obtained from Therapeutic Target Database (TTD, https://db.idrblab.org/ttd/), DrugBank Database (https://www.drugbank.ca/), Pharmacogenomics Knowledgebase (PharmGKB, https://www.pharmgkb.org/), Comparative Toxicogenomics Database (CTD, https://www.pharmgkb.org/), and DisGeNET (https://www.disgenet.org/search). The gene identifiers were also converted into official gene symbols using an R-script linking the website of PubMed (https://www.ncbi.nlm.nih.gov/gene/), and we only included human genes.

### 2.4. Constructing Drug-Target-Disease Network and Identifying Hub Genes

The common genes between the targets of active compounds and DR-related genes were extracted to construct the drug-target-disease network, and the interactions between genes were based on the STRING database (https://string-db.org/). The parameters of degree, betweenness, and closeness in topological analysis were calculated to assess the centrality of each gene node in the network, and the hub genes were chosen as key genes in the treatment of MFD for DR. The network was visualized by Cytoscape 3.4.0, and the analysis was conducted by its plugin of NetworkAnalyzer.

### 2.5. Molecular Docking

Molecular docking analysis was conducted to evaluate the binding affinity of the active compounds towards the protein of hub gene. Crystal structure of tumor necrosis factor (TNF, PBD ID: 2AZ5) in complex with the small molecular inhibitor of C_32_H_32_F_3_N_3_O_2_ (PDD ID: 307) was retrieved from the Protein Data Bank (PDB) Database (https://www.rcsb.org/). To optimize the protein structure, we extracted the ligand, removed water, charged termini, and added hydrogens by using SYBYL-X 2.0 (Certara Inc., USA). Then, after generating the protomol in the prepared protein, the compounds were docked, respectively, into the binding site present in the protomol by employing the Surflex-Dock module in the SYBYL software. The consensus score (C-score) was used to assess the binding affinity, and the compounds with high C-score of 4 or 5 were classified as good docked [12].

### 2.6. Diabetic Rat Model

Fifty Sprague-Dawley (SD) rats of 8∼10 weeks and 200∼250 grams were acquired from Beijing Weitong Lihua Experimental Animal Technology (Beijing, China). After one-week acclimatization, the rats were fed with a high-fat diet (HFD) for two weeks (Beijing Keao Xieli Feed, China) and then intraperitoneally injected with streptozotocin (STZ, 55 mg/kg) (Sigma, USA) to induce T2DM. Some rats were served as normal control by feeding with the standard diet and injecting with physiological saline. The level of fasting blood glucose (FBG) was measured 3 days after the injection, and the rats with FBG ≥16.7 mmol/L were considered as diabetic rats. The blood samples were collected from the tail vein. All experimental procedures and protocols were approved by the Ethics Committee of Eye Hospital of Wenzhou Medical University (2018016).

### 2.7. Treatment Protocols

The rats with FBG ≥16.7 mmol/L were randomly divided into three groups: (1) treated with physiological saline by intragastric gavage (DR group); (2) treated with low-dose MFD (5.9 g/kg·d, equivalent to 5 times the dose in humans, twice/day for 3 months) by intragastric gavage (low-dose MFD group); (3) treated with high-dose MFD (11.7 g/kg·d, equivalent to 10 times the dose in humans, twice/day for 3 months) by intragastric gavage (high-dose MFD group). The herbs in MFD were purchased from Fujian Medicinal Materials (Fuzhou, China). After boiling in water for 30 min, the decoction was filtered and collected.

### 2.8. Biochemical Measurements

The rats were anesthetized after the treatment, and blood samples were collected from the abdominal aorta. After 3000 rpm centrifugation for 10 min at 4°C, the serum was collected to measure FBG, aspartate aminotransferase (AST), and glutamic-pyruvic transaminase (ALT) using the automatic biochemical analyzer of AU480 (Beckman, USA) according to the kit instructions.

### 2.9. Retinal Histological Assessment

Retinas were fixed in 4% paraformaldehyde solution after isolation. Then, retinas were sectioned (5 *μ*m), stained with hematoxylin and eosin (H&E), and observed under the microscope (Olympus, Japan).

### 2.10. Western Blot Analysis

Nuclear and cytosolic proteins in retinas were isolated as described in the extraction kits (BestBio Science, China). The protein concentration in each sample was normalized to the equal protein concentration. The protein samples were separated by SDS-PAGE (Sigma, USA) and then electrophoretically transferred onto the PVDF membrane (Millipore, USA). The membranes were incubated with primary and secondary antibodies (Abcam, UK). Immunoblots were visualized by the enhanced chemiluminescent kits (Sigma, USA). The grey densities of the protein bands were normalized by using *β*-actin or GAPDH density as an internal control, respectively.

## 3. Results

### 3.1. Identification of Active Compounds and the Targets and DR-Related Genes

**Figure 2:**
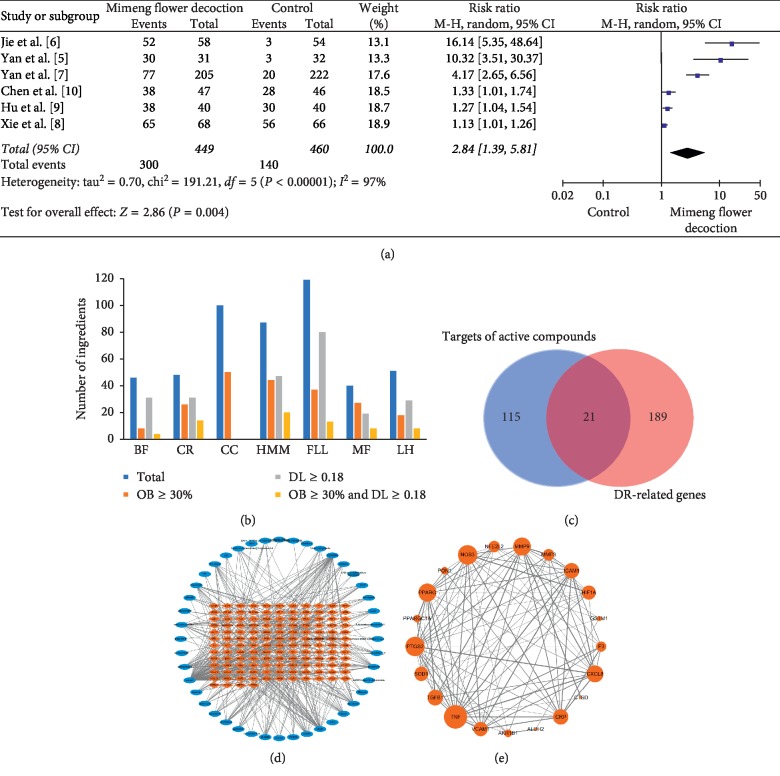
Systems pharmacology analysis of Mimeng flower decoction (MFD) in treating diabetic retinopathy (DR). (a) Meta-analysis of the efficacy of MFD in treating DR. (b) Screening active compounds in MFD. (c) Network of active compounds and the targets. Blue nodes represent active compounds, and orange nodes represent potential targets. The edge represents the interaction between them. (d) Overlap analysis of the targets of active compounds and DR-related genes. (e) Compound-target-disease network. The nodes represent the genes, and node size is proportional to its degree. The edges represent the interaction between genes, and edge size is proportional to the interaction score based on the STRING database. DL, drug-likeness; OB, oral bioavailability.

In the pooled analysis, six clinical trials were reported an obvious efficacy of MFD in treating DR with a total of 449 MFD-treated patients and 460 controls, and the overall estimate of relative risk (RR) was 2.84 (95% confidence interval (CI): 1.39∼5.81) (Figure 2(a)) [5–10].

Then, ADME parameters were used to identify the potential active compounds in MFD, which consisted of seven herbs: *Buddlejae Flos* (BF), *Coptidis Rhizoma* (CR), *Cinnanmomi Cortex* (CC), *Hedysarum Multijugum Maxim.* (HMM), *Fructus Ligustri Lucidi* (FLL), *Mume Fructus* (MF), and *Leonuri Herba* (LH). According to the criteria of DL ≥ 0.18 and OB ≥ 30%, a total of 57 active compounds were identified (BF (4), CR (14), CC (0), HMM (20), FLL (13), MF (8), and LH (8)) (Figure 2(b); Table 1; [Supplementary-material supplementary-material-1]).

Subsequently, these active components were used as bait to fish for the targets in TCMSP. After normalizing the gene names and removing duplication, we finally screened out a total of 136 targets (Figure 2(d); [Supplementary-material supplementary-material-1]). The DR-related genes were searched in the databases of DrugBank, TTD, PharmGKB, CTD, and DisGeNET. After normalizing the gene symbols and removing duplication, we finally identified 210 DR-related genes.

### 3.2. Construction of Component-Target-Disease Network and Hub Gene Identification

**Figure 3:**
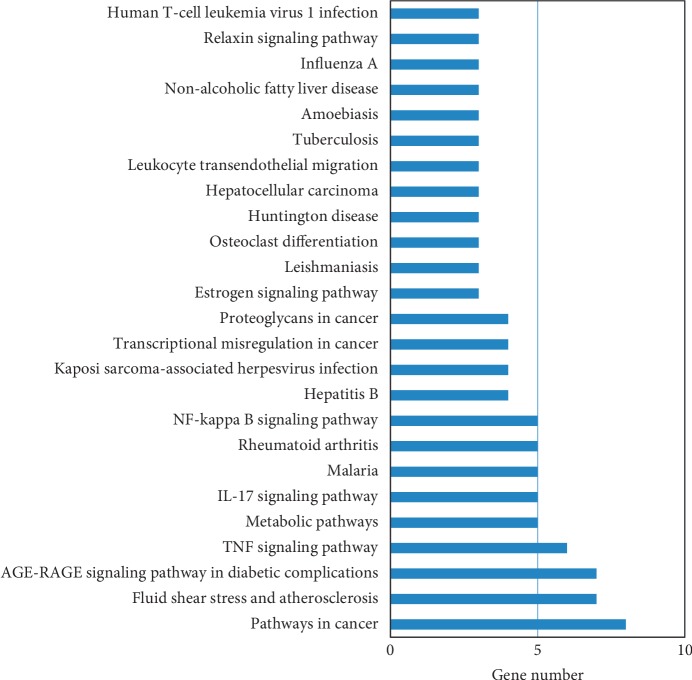
KEGG pathway analysis of the genes in the compound-target-disease network.

Twenty-one common genes between the targets of active compounds and DR-related genes were selected to construct the compound-target-disease network by employing the protein-protein interaction (PPI) (Figures 2(c) and 2(e)). These genes were involved in multiple KEGG pathways, including AGE-RAGE signaling pathway in diabetic complications and TNF signaling pathway (Figure 3). In topological analysis of the network, tumor necrosis factor (TNF) had a significantly higher degree, closeness, and betweenness centrality than other gene nodes (Table 2). Thus, TNF was identified as the hub gene.

### 3.3. Binding Affinity of the Active Compounds towards TNF

**Figure 4:**
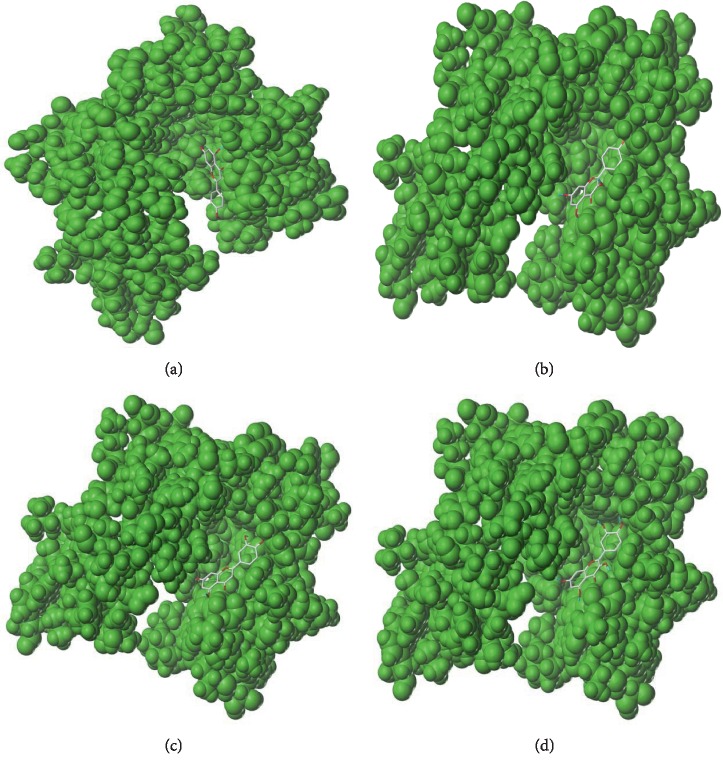
Molecular docking analysis of the binding affinity of active compounds (acacetin, kaempferol, luteolin, and quercetin) towards the target of tumor necrosis factor (TNF). C-score, consensus score. (a) Acacetin C-score = 4; (b) kaempferol C-score = 4; (c) luteolin C-score = 4; (d) quercetin C-score = 4.

In the previous analysis, TNF was targeted by four active compounds in MFD (acacetin, kaempferol, luteolin, and quercetin). To investigate whether these compounds could interact with TNF directly or not, molecular docking simulation of the interaction was conducted, respectively (Figure 4). Acacetin could form one hydrogen bond with the residue A/TYR151 of the TNF protein and showed a good binding affinity towards TNF (C-score = 5). Kaempferol could form two hydrogen bonds with the residues A/TYR151 and B/TYR151 and showed a good binding affinity (C-score = 4). Luteolin could form four hydrogen bonds with the residues A/TYR151, B/TYR151, A/GLY121, and A/GLY122 and showed a good binding affinity (C-score = 4). Quercetin could form two hydrogen bonds with the residues A/TYR151 and B/TYR151 and showed a good binding affinity (C-score = 4).

### 3.4. Efficacy of MFD in Treating Diabetic Rats

**Figure 5:**
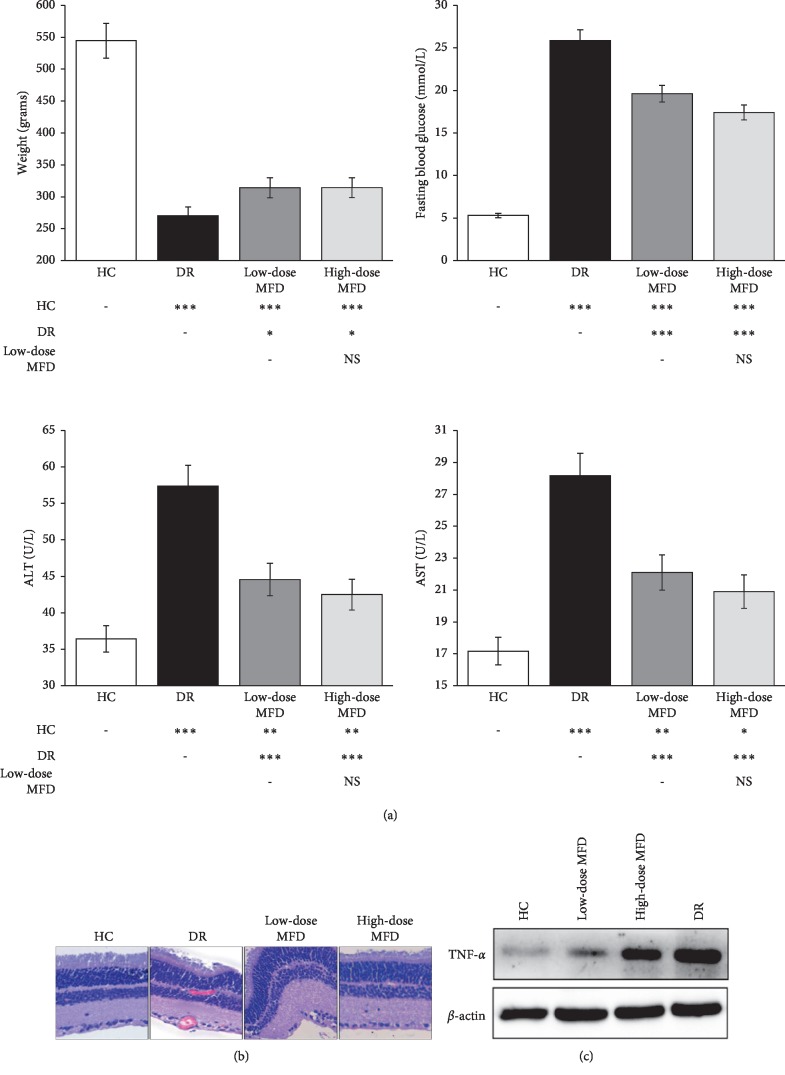
Efficacy of Mimeng flower decoction (MFD) in treating diabetic rats and its effect on the retinal expression of tumor necrosis factor (TNF). (a) Effects of MFD treatment on body weight, fasting blood glucose, ALT, and AST in diabetic rats (*n* = 10 per group). (b) Retinal histological assessment of MFD treatment in diabetic rats. (c) Effects of MFD treatment on retinal TNF expression in diabetic rats. DR, diabetic retinopathy; HC, healthy control. ^*∗*^*P* < 0.05, ^*∗∗*^*P* < 0.01, ^*∗∗∗*^*P* < 0.01.

STZ-induced diabetic rats (*n* = 10) had a significant decrease in body weight (*P* < 0.001) and a significant increase in FBG, ALT, and AST (*P* < 0.001) when compared with the healthy controls (HCs) (*n* = 10) (Figure 5(a)). After the administration of MFD for 3 months, the rats in both low-dose and high-dose MFD groups had a significant increase in body weight (*P* < 0.05) and a significant decrease in FBG, ALT, and AST (*P* < 0.001) when compared with the diabetic rats (*n* = 10 per group). No significant difference was detected between the low-dose and high-dose MFD groups (*P* > 0.05). These results indicated the hypoglycemic and hepatoprotective effects of MFD in treating diabetes.

In retinal histological assessment, the diabetic rats had a larger number of vessels in the ganglion cell layer (GCL), inner nuclear layer (INL), and outer nuclear layer (OPL), and a thinner retina, GCL, and nerve fiber layer (NFL) than the HCs (Figure 5(b)). After MFD treatment, the vessel number decreased and the retinal thickness increased, but no significant difference was detected between the low-dose and high-dose MFD groups. This indicated the efficacy of MFD in treating DR.

### 3.5. Effects of MFD Treatment on Retinal TNF Expression in Diabetic Rats

The diabetic rats had a higher retinal expression of TNF than the HCs (*P* < 0.05) (Figure 5(c)). After MFD treatment, TNF expression decreased significantly (*P* < 0.05), but no obvious difference was detected between the low-dose and high-dose MFD groups (*P* > 0.05).

## 4. Discussion

DR is one of the most serious complications in DM, and it is characterized by blood stasis and the deficiency of “qi” and “yin” according to the theory of TCM. MFD has a good performance in invigorating blood circulation and tonifying “qi” and “yin,” and thus, it was thought to be effective in treating DR. In our meta-analysis of clinical trials, it showed a good efficacy in DR treatment. However, the molecular mechanism is still unclear. As a complex of several herbs, we do not know which ingredient works and the potential targets.

To explore the mechanism, we adopted the methods of systems pharmacology. First, active compounds were filtered out and used as bait to fish potential targets. The DR-related genes were obtained by the systematic search. Finally, 21 common genes were identified to construct the compound-target-disease network. Among the 21 genes, multiple genes have been reported in the pathogenesis of DR. For example, the genotypes of NOS3 rs869109213 polymorphism were associated with the risk of DR [13]. High glucose-induced hyperosmolarity promoted PTGS2 (COX-2) expression and angiogenesis [14]. MMP9 was implicated in retinal capillary cell apoptosis and subsequent development of DR [15]. PPARG has been strongly indicated as a primary target in the treatment of DR [16].

In the KEGG pathway analysis, these genes were associated with multiple pathways, such as AGE-RAGE signaling pathway in diabetic complications, TNF signaling pathway, IL-17 signaling pathway, and NF-kappa B signaling pathway, indicating the involvement of inflammatory signaling pathways in the development of DR. These genes might be potential therapeutic targets for DR. It has been found that IL-17A could exacerbate DR by impairing Müller cell function, and blocking IL-17A could alleviate DR [17, 18].

In topological analysis of the compound-target-disease network, TNF showed a significant network centrality than other gene nodes, and it was identified as the hub gene. TNF-*α* has been demonstrated to be involved in a variety of intraocular inflammatory diseases via the proinflammatory effects [19, 20]. Furthermore, the active compounds in MFD showed a good binding affinity towards TNF, indicating that MFD might exert the therapeutic role by targeting TNF.

In an animal experiment, MFD showed a good efficacy in treating diabetic rats and reversing retinal impairment, which was consistent with the clinical trials. The TNF expression also decreased significantly after MFD treatment, but no dose-response relationship was found. This might contribute to the dose interval or treatment duration which was not large or long enough to magnify the difference. In the clinical study of Xia et al., fundus fluorescein angiography (FFA) found a significant decrease of new vessels among the patients supplemented with one-month or two-month MFD treatment when compared with the control, but no obvious difference was detected between the one-month and two-month groups (effective rates: 89.7% *vs.* 95.6%, *P* > 0.05) [8]. Similarly, in the clinical study of Chen et al., optical coherence tomography (OCT) detected a significant decrease of macular edema in the MFD treatment groups, but no obvious difference was detected between the one-month and two-month groups (314.57 ± 109.51 *vs.* 308.64 ± 130.57, *P* > 0.05) [10]. Thus, we thought the effective dose of MFD was easily available, but it might need several times the effective dose to reach a better efficacy, which was not recommended considering the adverse effects (Figure 5).

## 5. Conclusion

This study adopted the method of systems pharmacology to screen out active compounds and construct the compound-target-disease network and found that MFD could ameliorate DR by downregulating the network hub gene of TNF.

## Figures and Tables

**Table 1:** Active compounds in Mimeng flower decoction.

Mol ID	Molecular weight	Oral bioavailability (%)	Drug-likeness
MOL000006	286.25	36.16	0.25
MOL000033	428.82	36.23	0.78
MOL000098	302.25	46.43	0.28
MOL000211	456.78	55.38	0.78
MOL000239	314.31	50.83	0.29
MOL000296	414.79	36.91	0.75
MOL000354	316.28	49.60	0.31
MOL000358	414.79	36.91	0.75
MOL000371	314.36	53.74	0.48
MOL000374	642.67	41.72	0.69
MOL000378	316.38	74.69	0.30
MOL000379	462.49	36.74	0.92
MOL000380	300.33	64.26	0.42
MOL000387	418.38	31.10	0.67
MOL000392	268.28	69.67	0.21
MOL000398	316.33	109.99	0.30
MOL000417	284.28	47.75	0.24
MOL000422	286.25	41.88	0.24
MOL000433	441.45	68.96	0.71
MOL000438	302.35	67.67	0.26
MOL000439	626.67	49.28	0.62
MOL000442	314.31	39.05	0.48
MOL000449	412.77	43.83	0.76
MOL000622	266.37	63.71	0.19
MOL000762	510.52	35.36	0.65
MOL000785	352.44	64.60	0.65
MOL000953	386.73	37.87	0.68
MOL001040	272.27	42.36	0.21
MOL001418	376.54	61.02	0.38
MOL001420	412.77	38.00	0.76
MOL001421	334.50	85.97	0.33
MOL001422	334.50	66.29	0.33
MOL001439	304.52	45.57	0.20
MOL001454	336.39	36.86	0.78
MOL001458	320.34	30.67	0.86
MOL001689	284.28	34.97	0.24
MOL001790	592.60	39.84	0.71
MOL002668	334.37	45.83	0.87
MOL002894	322.36	35.74	0.73
MOL002897	336.39	43.09	0.78
MOL002903	339.42	55.37	0.77
MOL002904	351.38	36.68	0.82
MOL002907	404.55	104.95	0.78
MOL004576	304.27	57.84	0.27
MOL005043	400.76	37.58	0.71
MOL005146	568.63	48.87	0.71
MOL005147	406.47	54.41	0.47
MOL005169	486.86	40.23	0.82
MOL005190	288.27	71.79	0.24
MOL005195	450.48	83.12	0.80
MOL005209	401.60	30.11	0.75
MOL005211	696.87	65.45	0.23
MOL005212	404.55	103.23	0.78
MOL006673	468.84	46.04	0.83
MOL008601	318.55	46.90	0.23
MOL008647	313.38	86.71	0.26
MOL013352	454.56	43.29	0.77

**Table 2:** Topological analysis of the compound-target-disease network.

Gene symbol	Gene name	Degree	Closeness	Betweenness
TNF	Tumor necrosis factor	18	0.91	0.15
NOS3	Nitric oxide synthase 3	15	0.80	0.05
PTGS2	Prostaglandin-endoperoxide synthase 2	15	0.80	0.05
MMP9	Matrix metallopeptidase 9	14	0.74	0.05
PPARG	Peroxisome proliferator-activated receptor-gamma	14	0.74	0.03
CXCL8	C-X-C motif chemokine ligand 8	13	0.71	0.02
ICAM1	Intercellular adhesion molecule 1	12	0.69	0.02
CRP	C-reactive protein	12	0.69	0.02
TGFB1	Transforming growth factor beta 1	12	0.67	0.01
SOD1	Superoxide dismutase 1	11	0.69	0.06
HIF1A	Hypoxia-inducible factor 1 subunit alpha	11	0.67	0.01
VCAM1	Vascular cell adhesion molecule 1	11	0.65	0.00
NFE2L2	Nuclear factor, erythroid 2 like 2	9	0.65	0.04
MMP3	Matrix metallopeptidase 3	9	0.61	0.00
F3	Coagulation factor III, tissue factor	8	0.59	0.00
AKR1B1	Aldo-keto reductase family 1 member B	6	0.59	0.07
PON1	Paraoxonase 1	6	0.59	0.02
PPARGC1A	PPARG coactivator 1 alpha	6	0.57	0.00
GSTM1	Glutathione S-transferase mu 1	4	0.48	0.02
ALDH2	Aldehyde dehydrogenase 2 family member	2	0.41	0.00
CTSD	Cathepsin D	2	0.50	0.00

## Data Availability

The data used to support the findings of this study are available from the corresponding author upon request.

## Supplementary Materials

Supplementary Materials Table S1: Mimeng flower decoction comprising of seven herbs. Table S2: the active compounds in Mimeng flower decoction and their target genes.
